# Efficacy of High-Resolution Preoperative 3D Reconstructions for Lesion Localization in Oncological Colorectal Surgery—First Pilot Study

**DOI:** 10.3390/healthcare10050900

**Published:** 2022-05-12

**Authors:** Domenico Soriero, Paola Batistotti, Rafaela Malinaric, Davide Pertile, Andrea Massobrio, Lorenzo Epis, Beatrice Sperotto, Veronica Penza, Leonardo S. Mattos, Marina Sartini, Maria Luisa Cristina, Alessio Nencioni, Stefano Scabini

**Affiliations:** 1 General and Oncologic Surgery, IRCCS Ospedale Policlinico San Martino, 16132 Genoa, Italy; soriero.domenico@gmail.com (D.S.); rafaela.malinaric@gmail.com (R.M.); davperti@gmail.com (D.P.); massobrioandrea@gmail.com (A.M.); loreepis@gmail.com (L.E.); beatrice.sperotto@gmail.com (B.S.); stefanoscabini@libero.it (S.S.); 2 Department of Integrated Surgical and Diagnostic Sciences, University of Genoa, 16132 Genoa, Italy; paola.batistotti@gmail.com; 3 Urological Clinical Unit, San Martino Hospital, 16132 Genoa, Italy; 4 Biomedical Robotics Lab, Department of Advanced Robotics, Istituto Italiano di Tecnologia, 16163 Genoa, Italy; veronica.penza@iit.it (V.P.); leonardo.demattos@iit.it (L.S.M.); 5 Department of Health Sciences, University of Genoa, Via Pastore 1, 16132 Genoa, Italy; 6 Operating Unit Hospital Hygiene, Galliera Hospital, Mura delle Cappuccine 14, 16128 Genoa, Italy; 7 Section of Geriatrics, Department of Internal Medicine and Medical Specialties (DIMI), University of Genoa, 16132 Genoa, Italy; alessio.nencioni@unige.it; 8 Gerontology and Geriatrics, IRCCS Ospedale Policlinico San Martino, 16132 Genoa, Italy

**Keywords:** colon cancer, CT scan, localization

## Abstract

When planning an operation, surgeons usually rely on traditional 2D imaging. Moreover, colon neoplastic lesions are not always easy to locate macroscopically, even during surgery. A 3D virtual model may allow surgeons to localize lesions with more precision and to better visualize the anatomy. In this study, we primary analyzed and discussed the clinical impact of using such 3D models in colorectal surgery. This is a monocentric prospective observational pilot study that includes 14 consecutive patients who presented colorectal lesions with indication for surgical therapy. A staging computed tomography (CT)/magnetic resonance imaging (MRI) scan and a colonoscopy were performed on each patient. The information gained from them was provided to obtain a 3D rendering. The 2D images were shown to the surgeon performing the operation, while the 3D reconstructions were shown to a second surgeon. Both of them had to locate the lesion and describe which procedure they would have performed; we then compared their answers with one another and with the intraoperative and histopathological findings. The lesion localizations based on the 3D models were accurate in 100% of cases, in contrast to conventional 2D CT scans, which could not detect the lesion in two patients (in these cases, lesion localization was based on colonoscopy). The 3D model reconstruction allowed an excellent concordance correlation between the estimated and the actual location of the lesion, allowing the surgeon to correctly plan the procedure with excellent results. Larger clinical studies are certainly required.

## 1. Introduction

Colorectal lesion resection is one of the most frequently performed surgical procedures. The anatomy, especially of the right-sided colon, varies considerably from patient to patient [1,2]. Neoplastic lesions are not always easy to locate, and the lack of haptic feedback during minimally invasive surgery complicates their intraoperative identification [3,4]. These factors stress the importance of accurate preoperative planning, which is essential to reduce the risks of complications and to achieve the complete excision of suspected lesions. To this end, surgeons should know the precise location of the lesion in order to plan the access entry point on the abdomen, the incision for the lesion resection and the vascular structures that need to be ligated [5,6,7].

Although CT scanning and MRI are considered the gold standard for preoperative planning, they only provide a 2D visualization of patient anatomy, causing possible inconsistencies between what is preoperatively observed and mentally reconstructed by the surgeon and what is actually found intraoperatively [8]. However, the volumetric nature of the imaging based on body slicing allows for the conversion of the 2D diagnostic imaging to three-dimensional (3D) rendering and models [9], making the visualization of patient anatomy more intuitive. New technologies have also been proposed for the precise localization of neoplastic colonic lesions, such as *magnetic endoscopic imaging* studied by Miroslaw Szura et al. [10].

Currently, several surgical specialties are already exploiting 3D reconstruction models extracted from CT scanning and MRI during surgical planning; in orthopedics, for example, or urology, 3D models are used to identify kidney lesions or to improve the planning of robot-assisted radical prostatectomies [11,12,13,14]. In general surgery, 3D reconstruction has been demonstrated to be a valuable tool to help surgeons in designing a more effective surgical plan for the treatment of complex cases of esophagogastric junction pathologies [15]. 

Other studies consider the use of 3D surface rendering useful, especially in undiagnosed vessel anomalies, such as undiagnosed short arterial trunks that could lead to improper surgical decisions with higher intraoperative and postoperative hemorrhage risks or anastomosis leakage. For example, a case report, involving a patient with horseshoe kidney who underwent an anterior resection of rectosigmoid cancer, states the importance of evaluating gonadal vessels, sacral arteries, branches of hypogastric arteries and inferior mesenteric arteries, which were identified and preserved due to 3D model [15,16,17]. 

Moreover, in colorectal oncological surgery, even if the sensitivity of detecting colorectal lesions through CT imaging is extremely high, there are some cases where lesions can be missed and misdiagnosed [18,19].

This study presents an analysis of the efficacy of visualizing a high-resolution preoperative patient-specific 3D reconstruction during the planning of colorectal oncological surgeries. Two surgeons were asked to plan the surgery of fourteen patients with colorectal lesions with indication for radical surgery. One of them only had access to 2D images, while the other could visualize the patient-specific 3D reconstruction. The preoperative plans of both of the surgeons were then compared to each other and with intraoperative and histopathological findings. In this pilot study conducted at the Oncological Surgery Department of Scientific Institute for Research, Hospitalisation and Health Care (IRCCS), Hospital San Martino (Genova, Italy), ‘Hyper Accuracy 3D™’ (HA3D™, Medics 3D, Moncalieri, Italy) was used to create a 3D reconstruction from CT/MRI images and, if necessary, from colonoscopy.

## 2. Materials and Methods

### 2.1. Study Design

This is a monocentric, prospective, observational, pilot study, which includes 14 consecutive patients who presented colorectal lesions with surgical indication. The study started in July 2018 at the oncological surgery department of IRCCS Policlinico San Martino (Genova, Italy).

The primary aim of this study was to evaluate the efficacy of high-resolution preoperative 3D reconstructions in colorectal lesion localization tasks. In particular, this was achieved by evaluating the consistency between each surgeon’s preoperative plan and the intraoperative decisions and actions.

In Section 4, a descriptive study of 5 clinical cases was performed, choosing lesions localized in different parts of the colon and rectum. We tried to highlight the fundamental elements of our decision-making process.

### 2.2. Patient Selection

We selected 14 consecutive patients diagnosed with colorectal lesions and indication for treatment using radical surgery with curative intent in an elective setting. Each lesion was firstly diagnosed through colonoscopy and then confirmed by a CT/MRI scan. Patients who did not undergo a preoperative CT/MRI scan or had a CT/MRI scan that did not permit an adequate 3D model reconstruction (more than 1,5 mm slice thickness) were excluded from the study, as well as underaged patients. 

### 2.3. The 3D Reconstruction Software

**Figure 1:**
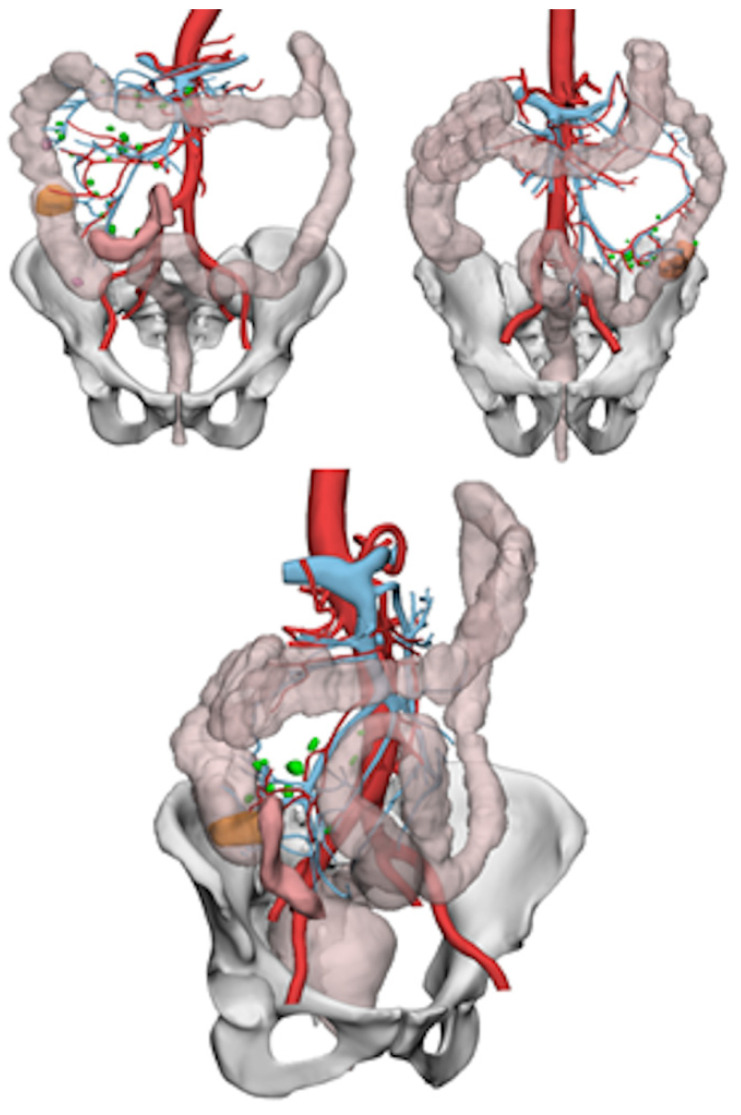
Example of 3D model reconstruction.

The HA3D™ service was used to generate patient-specific 3D models (Figure 1) using CT/MRI scans and, if necessary, combining this information with colonoscopy. HA3D™ technology integrates engineering modeling knowledge with clinical know-how in order to obtain 3D patient-specific medical-grade models based on 2D images. HA3D™ reconstructions can be manipulated by the surgeon, generating 3D model views that can be fitted, rotated, zoomed and virtually dissected, allowing a clearer study of the lesion itself. The reconstruction can also reveal possible anomalies near the neoplasm, such as inflammation, lymph nodes or smaller adjacent lesions.

The 3D models were available in two days. The acquired colonoscopy and CT/MRI scan data used were anonymized. 

### 2.4. Research Protocol

**Figure 2:**
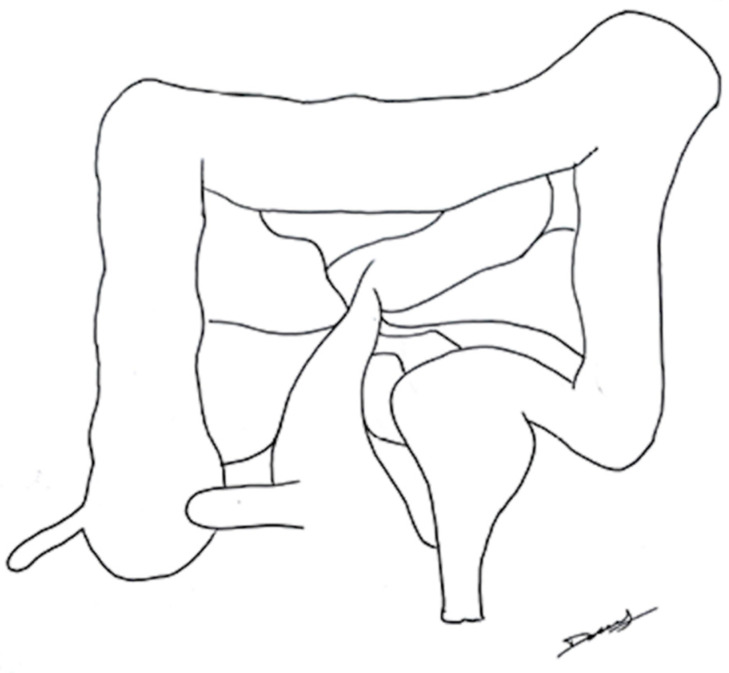
A simplified design of the colon.

Two surgeons, hereafter indicated as Surgeon A (SA) and Surgeon B (SB), were selected to perform the task for each surgical operation. Both of them have been working in a high-volume Colorectal Surgery Unit (each performing more than 50 procedures per year) and cooperating with each other for 15 years. During the study, they used the same action protocols and performed the interventions with the same techniques and indications. SA was selected to perform the interventions by pre-operatively analyzing the gold standard 2D radiological images, while SB had access to the pre-operative 3D model reconstructions and could plan the surgery according to them. The 3D reconstruction visualization was available for 15 min. Both surgeons were indicated to locate the lesion and display which kind of surgery they would have performed on a simplified design of the colon (Figure 2). SB did not influence SA’s intervention in any way.

In order to facilitate the description of the surgical actions, the colon was divided into 4 different surgical segments: the right-sided colon (caecum, ascending colon and proximal transverse colon), transverse colon (medial and distal part, including the splenic flexure), left-sided colon (descending and sigmoid colon) and rectum. They correspond to typical surgical sites of oncological colorectal surgery. 

To date, 3D reconstruction imaging has not been validated for pre-operative study in colorectal cancer; therefore, it was necessary for SA to not see the images pre-operatively in order to avoid conditioning. As a matter of fact, this would have made the study experimental, thus putting the good clinical practice at risk. 

After the surgical operation, the following evaluations were made:

- **SB planning vs. intraoperative and histopathological findings:** The localization of the lesion performed by SB (based on the 3D models) was compared with intraoperative and histopathological findings (macroscopic characteristics).
- **SB planning vs. SA planning:** The localization of the lesion performed by SB was compared with SA’s 2D image-based localization.
- **SB planning vs. SA surgical procedure:** SB’s surgical pre-operative planning was compared with the intra-operative surgical procedure performed by SA.

This study was approved by the Ethical Committee of the Liguria region (Italy) on 16 December 2019 with protocol number [345/2019].

### 2.5. Statistical Analysis

Data are expressed as means and standard deviation or through frequency distribution. Cohen’s kappa coefficient was used to assess the inter-agreement between the data collected with the 3D System, with the CT and during surgery. Analysis was performed using STATA/SE14tm software (StataCorp LP, College Station, TX, USA).

### 2.6. Study Limitations

✓ Limited number of cases;
✓ Limited number of surgeons who have tested the software;
✓ No specific training of surgeons in viewing radiological images;
✓ The prospective observational nature of the study.

## 3. Results

The study was conducted between July 2018 and July 2019 and involved 14 patients. All patients were Caucasian. The mean age was 74 ±11.5 years old; six patients were male and eight were female. There were no absolute contraindications for performing laparoscopic procedures. The postoperative histopathological findings revealed 12 malignant lesions and 2 benign lesions. 

The mean time that elapsed between surgery and preoperative staging was 9 days (STD 9.5). Among the 14 patients, 7 patients (50%) had a right-sided colon lesion (caecum, ascending colon and proximal transverse colon), 3 (21.43%) had a rectal lesion, 2 (14.28%) left-sided (descending and sigmoid colon), and 2 (14.28%) had a transverse colon lesion (medial and distal transverse colon and splenic flexure) documented on the CT scan. Two patients (14.28%) had a suspected right-sided colon lesion shown only during colonoscopy but not evidenced by the CT scan. In order to reconstruct the 3D models for these patients, both colonoscopy and CT scan were used. 

**Figure 3:**
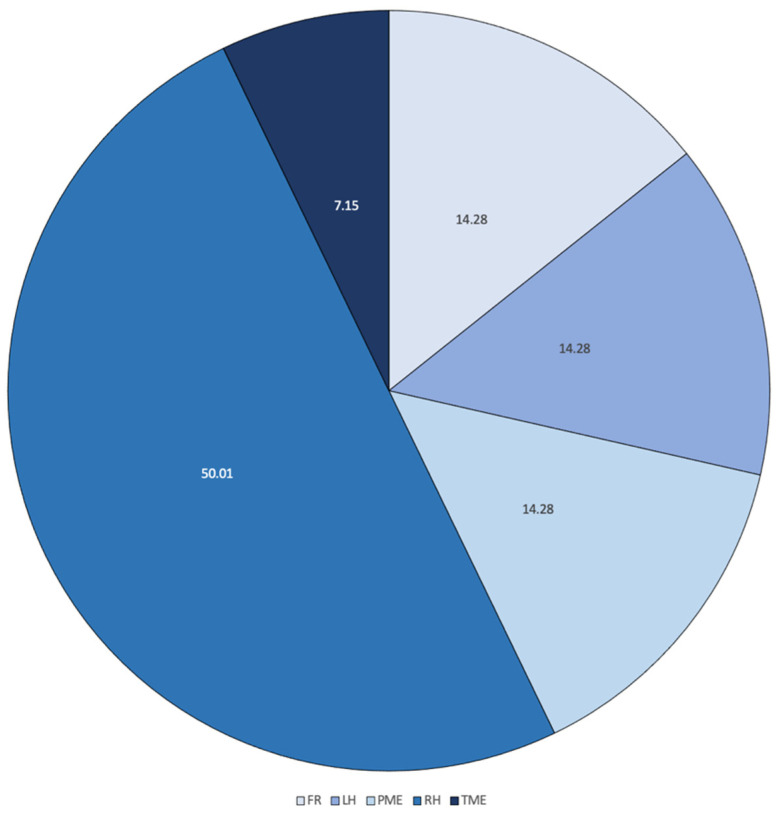
Frequency distribution of different types of resections performed on patients (FR: flexure resection, LH: left hemicolectomy, RH: right hemicolectomy, PME: partial mesorectal excision, TME: total mesorectal excision).

Based on the pre-operative identification of the suspected lesion location, patients underwent the indicated surgical procedures according to standard clinical practice (right hemicolectomy, left hemicolectomy, transverse, splenic flexure and total or partial mesorectal resection) (Figure 3), showing vessel ligation.

When comparing the lesion localizations identified on the 3D models and the histopathological findings, a 100% concordance correlation was found (observed agreement 100%, Kappa 1). 

**Figure 4:**
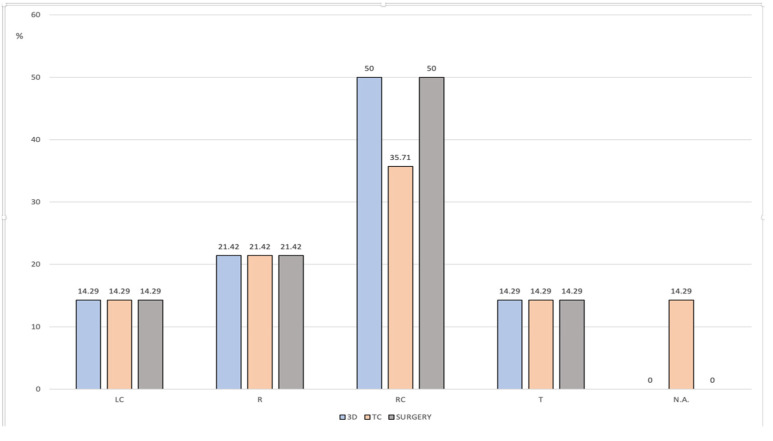
Frequency distribution of tumor localization based on 3D reconstruction, CT/MRI scans and intraoperative findings. (LC: left colon, RC: right colon, R: rectum, T: transverse colon, N.A.: not evident).

This was not true, however, for the comparison between the conventional method based on the 2D CT scans and the visualization of the 3D models (observed agreement 85.7%, Kappa 0.8056). The errors were related to the two patients (14.29%) for whom the lesion was not evidenced on the CT scan, and the colonoscopy findings had to be used to plan the procedure (Figure 4).

## 4. Case Descriptions

### 4.1. Case 1

A 65-year-old female patient. 

History: hypertension, hypothyroidism and previous appendectomy and hysterectomy for benign pathology.

After multiple sub-occlusive episodes, she underwent colonoscopy, which revealed a sigmoid tumor that could not be crossed by the endoscopic instrument.

The histological examination of the biopsies harvested during colonoscopy was positive for adenocarcinoma.

The disease was staged T3N1M0 at the CT enema. The tumor was located in the proximal portion of the sigmoid colon.

According to this evidence, left video laparoscopic (VL) hemicolectomy surgery was scheduled.

**Figure 5:**
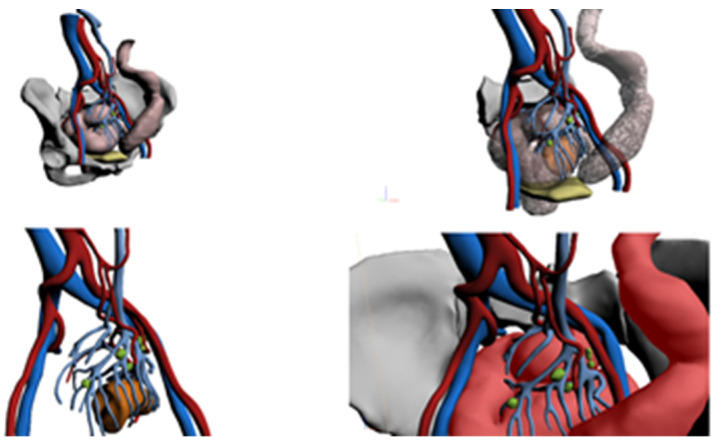
3D reconstruction (Case 1).

The 3D reconstruction showed the following (Figure 5):

- Substenosing neoplasm located in the distal portion of the sigma in a dolichosigma framework.
- Vascularization of the neoplasm came from the sigmoid arterial branches, which individually originate below the outlet of the left colic artery. This one was of moderate length.
- Suspicious lymph nodes were evident in the area surrounding the neoplasm only.

Because of this evidence, the surgery designed on the basis of 3D reconstructions was left hemicolectomy with preservation of the left colic artery.

During the surgery, the neoplasm was located in the distal sigma. Left hemicolectomy was then performed with left colic artery preservation and Knight–Griffen anastomosis without the need for splenic flexure mobilization.

### 4.2. Case 2

A 79-year-old female patient. No comorbidities. She came to our attention for SOF +, weight loss of about 10 kg in recent months and tenesmus. On colonoscopy imaging, a vegetative lesion was found about 17 cm from the anal rhyme involving the lumen circumferentially, not crossable by the endoscope. CT confirmed the presence of a large lesion in the sigma rectum, with thickening of the adjacent adipose tissue probably due to infiltration; multiple lymph nodes were noticed in the mesorectum, in the presacral adipose tissue, in the Douglas and in the iliac stations bilaterally. 

**Figure 6:**
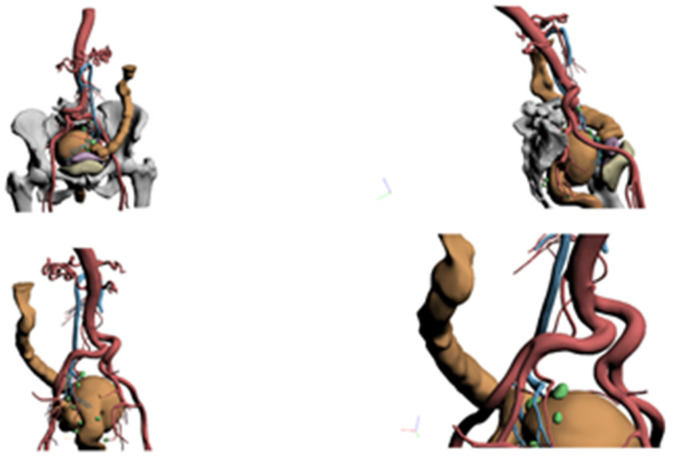
3D reconstruction (Case 2).

The 3D reconstruction highlighted the following (Figure 6):

- A large lesion of the sigma rectum joint, occupying a large portion of the pelvic excavation and in close contact with the contiguous organs (especially the uterus and bladder) and the iliac vessels, without signs of infiltration.
- The vascular reconstruction highlighted the inferior mesenteric artery, while the left colic artery was not visible.
- The presence of pathological lymph nodes in the Douglas and in the iliac area, especially on the left.

The patient underwent surgery with a robotic approach, subsequently converted to median laparotomy due to unclear cleavage plans of the neoplasm with the uterus and bladder. A partial mesorectal excision was then performed, with ligation of the inferior mesenteric vessels at the origin. A left colostomy was created. 

The final histological evaluation of the neoplasm described a pT4b/G3/N0 lesion.

### 4.3. Case 3

A 73-year-old female patient.

History: hypertension, treated with new oral anticoagulants (NOAs) for previous thrombophlebitis; previous appendectomy; and two caesarean sections.

She was admitted to the hospital for asthenia and anemia. Esophagogastroduodenoscopy (EGDS) and colonoscopy were performed: the latter revealed an adenocarcinoma of the right colon. A CT scan for the staging of the neoplasm followed. The findings were a hyperemic thickening of the walls of the ascending colon, inhomogeneity of the adjacent adipose layers, edema and millimeter mesenteric lymph nodes with extraserous involvement.

**Figure 7:**
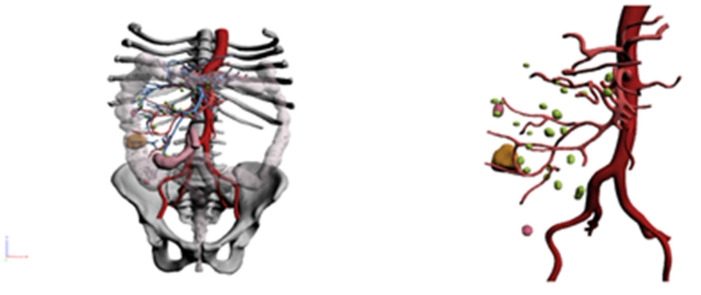
3D reconstruction (Case 3).

The 3D reconstructions showed the following (Figure 7):

- A neoplastic lesion located between the proximal and middle third of the ascending colon.
- Vascularization was ensured by the right ileocolic and colic arteries.
- Multiple mesenteric lymph nodes were noticed.

The patient underwent a right VL hemicolectomy with the ligation and section of the ileocolic vessels and of the middle colic artery right branch.

The histological examination staged a pT2/G2/N0 lesion.

### 4.4. Case 4

A 79-year-old male patient.

History: hypertension and previous ischemic heart disease: triple coronary artery bypass graft (CABG). 

He was investigated for severe anemia (6.9 g/L) and asthenia. He underwent endoscopic investigations; colonoscopy found a vegetative and stenosing lesion, not crossable by the endoscopic instrument and localized in the distal splenic flexure/distal transverse colon. Biopsies were collected. A CT scan was performed: it showed parietal thickening and a mass protruding the lumen with a large base causing substenosis of the lumen. 

**Figure 8:**
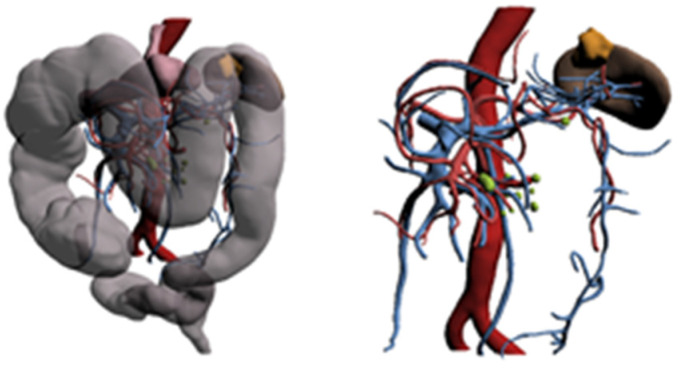
3D reconstruction (Case 4).

The 3D reconstructions showed the following (Figure 8):

- A heteroplastic lesion of the splenic flexure in the setting of a dolichocolon.
- Vascularization by the middle colic artery.
- Some mesenteric lymph nodes.

The patient underwent colic resection from the left colic angle to the proximal descending colon after the isolation and sectioning of the left colic and the left branch of the middle colic. 

Histological evaluation: pT4a/G2/N0 (for effracted and outdated serosa).

### 4.5. Case 5

An 80-year-old male patient.

History: hypertension, Chronic obstructive pulmonary disease (COPD) and pulmonary emphysema, duodenitis and previous appendectomy. He came to attention for alternate alvus. Investigations revealed a substenosing cuff lesion of the rectum. He underwent neoadjuvant therapy. 

The abdominal CT showed thickening of the rectal wall projecting into the lumen and resulting in substenosis extending to the anal rim. A few perirectal lymph nodes were present.

The lesion was reduced in size, extension and thickness on post-neoadjuvant MRI.

Surgery performed with a robotic approach was therefore planned. Skeletonization of the sigmoid and rectum, and TME with NS, Knight–Griffen anastomosis and a protective ileostomy were performed.

**Figure 9:**
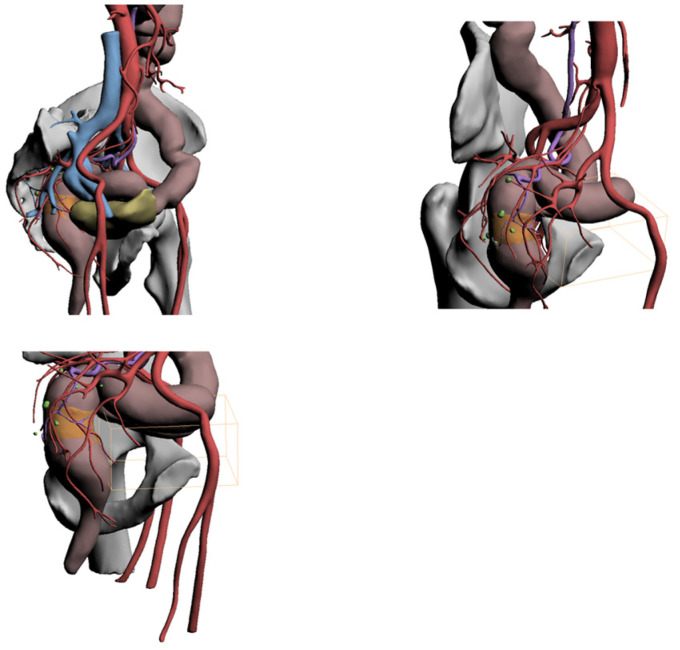
3D reconstruction (Case 5).

The 3D reconstruction displayed the following (Figure 9):

- Voluminous neoplastic mass of the middle rectum.
- Vascularization by the inferior mesenteric artery.
- Some locoregional lymph nodes.

Histological evaluation: ypt3/Gx/N0.

## 5. Discussion

This study performed an evaluation of the efficacy of using patient-specific 3D model reconstruction during preoperative planning in colorectal lesion localization. As mentioned earlier, colonoscopy and conventional imaging have some limitations when used in preoperative planning. For example, the lesion can be too small to appear on a CT scan, or vascular structures may be misrepresented [5]. Moreover, when a tumor does not involve the sierosa, it is invisible in an intraoperative setting, making the localization more difficult when using a laparoscopic approach due to the lack of tactile feedback [3,4]. The use of endoscopic clips [18] and peritumoral submucosal tattooing [20] can allow tumor localization at the time of surgery. However, these methods are invasive, and there is the risk of failure of lesion localization because of the dislocation of the clip and tattoo spread in the peritoneal cavity, in the superficial mucosa or in the mesenteric side. In contrast, our system, integrating endoscopic and radiological imaging, is not invasive and provides tumor localization before surgery.

Miroslaw Szura et al. localized colon lesions before surgery using *magnetic endoscopic imaging* (MEI) [8], stating that MEI allows a more accurate localization of the neoplastic infiltrate within the large intestine compared to standard colonoscopy alone.

In our study, the TC scan alone was not sufficient to localize two lesions because of their small dimensions, while colonoscopy correctly localized all the lesions. These two patients presented right-sided, malignant lesions. Such a situation can lead to risks, for example, the choice of an incorrect surgical approach or type of resection, leading to an incomplete excision.

Using ‘Hyper Accuracy 3D™’ (HA3D™, Medics, Moncalieri, Italy)’ software, which incorporates colonoscopy findings with CT abdominal scans, such lesions were not only evidenced but also studied more efficiently. 

Guerriero et al. [5] found an extremely short left colic artery trunk originating from the inferior mesenteric artery undiagnosed on a CT scan but documented on a 3D model. This finding can be compared to our undiagnosed right-sided colic lesions, demonstrating the importance of a preoperative 3D reconstruction of lesions to perform excisions with a high level of accuracy, without causing any damage.

Other studies focused primarily on lesion vascularization, as well as the vessels of organs found in the proximity. Some researchers found utility in the implementation of 3D surface rendering, especially in undiagnosed vessel anomalies, such as undiagnosed short arterial trunks that could lead to improper surgical decisions with higher intraoperative and postoperative hemorrhage risks or anastomosis leakage. Moreover, one case report involving a patient with horseshoe kidney, who underwent an anterior resection of rectosigmoid cancer, states the importance of evaluating gonadal vessels, sacral arteries, branches of hypogastric arteries and inferior mesenteric arteries, which, in this particular case, were identified and preserved due to 3D model SR [5,21,22]. 

Although our study is one of the first to evaluate the impact of using 3D models in colorectal surgery, it presents some limitations, the most important being related to the number of patients enrolled. However, we believe that this number is enough considering that our results are comparable to those of other published studies that tested 3D software reconstruction, even though the parameters taken into consideration are different.

## 6. Conclusions

This study provides a positive evaluation of the efficacy of high-resolution preoperative 3D reconstructions for the localization of colorectal lesions. The 3D models, built using both CT scans and colonoscopy data, were demonstrated to not be inferior to conventional 2D CT scans in tumor localization and pre-operative surgical planning. They could present good potential to improve lesion localization and help surgical planning, especially in difficult cases. We believe that this study will not only play a fundamental role in the pre-operative planning of surgery but also in 3D reconstructions’ application in intraoperative surgical guidance (e.g., based on AR/VR technologies) [23]. This study confirms that there is a reliability of 3D graphic images in the representation of reality, even in abdominal surgery. Further studies are certainly needed. 

## Data Availability

The data presented in this study are available on request to the corresponding author.
